# Resting State Glucose Utilization and Adult Reading Test Performance

**DOI:** 10.3389/fnagi.2020.00048

**Published:** 2020-03-05

**Authors:** Younghwa Lee, Dahyun Yi, Eun Hyun Seo, Ji Young Han, Haejung Joung, Min Soo Byun, Jun Ho Lee, Jongho Jun, Dong Young Lee

**Affiliations:** ^1^Interdisciplinary Program of Cognitive Science, Seoul National University, Seoul, South Korea; ^2^Institute of Human Behavioral Medicine, Medical Research Center, Seoul National University, Seoul, South Korea; ^3^Premedical Science, College of Medicine, Chosun University, Gwangju, South Korea; ^4^Department of Neuropsychiatry, Seoul National University Hospital, Seoul, South Korea; ^5^Department of Neuropsychiatry, Seoul National University Bundang Hospital, Sungnam, South Korea; ^6^Department of Geriatric Psychiatry, National Center for Mental Health, Seoul, South Korea; ^7^Department of Linguistics, Seoul National University, Seoul, South Korea; ^8^Department of Psychiatry and Behavioral Science, Seoul National University College of Medicine, Seoul, South Korea

**Keywords:** adult reading test, cerebral glucose metabolism, cognitive reserve, beta-amyloid, cognitively normal adults

## Abstract

Adult reading tests (ART) have been widely used in both research and clinical settings as a measure of premorbid cognitive abilities or cognitive reserve. However, the neural substrates underlying ART performance are largely unknown. Furthermore, it has not yet been examined whether the neural substrates of ART performance reflect the cortical regions associated with premorbid intelligence or cognitive reserve. The aim of the study is to identify the functional neural correlates of ART performance using 18F-fluorodeoxyglucose (FDG) positron emission tomography (PET) imaging in the cognitively normal (CN) middle- and old-aged adults. Voxel-wise analyses revealed positive correlations between glucose metabolism and ART performance in the frontal and primary somatosensory regions, more specifically the lateral frontal cortex, anterior cingulate cortex and postcentral gyrus (PCG). When conducted again only for amyloid-β (Aβ)-negative individuals, the voxel-wise analysis showed significant correlations in broader areas of the frontal and primary somatosensory regions. This is the first neuroimaging study to directly demonstrate the cerebral resting-state glucose utilization associated with ART performance. Our findings provide important evidence at the neural level that ART predicts premorbid general intelligence and cognitive reserve, as brain areas that showed significant correlations with ART performance correspond to regions that have been associated with general intelligence and cognitive reserve.

## Introduction

Estimation of premorbid cognitive ability is essential in both research and clinical settings to quantify and diagnose cognitive impairment. The most widely applied approach to estimating premorbid cognitive function is the use of an adult reading test (ART), which comprises of oral reading of orthographically irregular words. Premorbid intelligence can be estimated from ART based on the rationale that the reading ability of irregular word correlates strongly with measure of IQ in healthy adults (Nelson and Willison, 1991; Crawford et al., 2001; Yi et al., 2017), and is relatively resistant to cognitive declines in patients with neurological or psychiatric disorders (Sasanuma et al., 1992; McGurn et al., 2004; Matsuoka et al., 2006). It is assumed that better performance on ART implies prior knowledge of a word’s pronunciation and therefore a higher premorbid intelligence (Lezak et al., 2004). Moreover, numerous studies on Alzheimer’s disease (AD) have reported that an ability to pronounce irregular words is a retained skill even in advanced stages of the disease (Sasanuma et al., 1992; McGurn et al., 2004; Matsuoka et al., 2006), suggesting that ART is able to provide a reasonable estimate of premorbid intelligence for AD patients.

ART has also been used as an index for cognitive reserve (CR)—a theoretical concept referring to the cognitive capacity to cope with brain damages (Stern et al., 2003; Lo and Jagust, 2013; Rentz et al., 2017). The concept of CR has been widely used to explain the discrepancy between the clinical manifestations and degree of brain damage or pathology (Stern et al., 2003, 2008). It is also considered as a contributing factor toward individual differences of resilience to brain pathology. In epidemiologic studies, higher intelligence has consistently been shown to be protective against the progression of AD (Snowdon et al., 1996; Whalley and Deary, 2001; Yeo et al., 2011). Moreover, high prevalence of AD-related abnormal biomarkers at a given level of cognitive performance was associated with higher scores on ART, indicating that those with higher ART scores cope better with AD pathology (McGurn et al., 2004; Vemuri et al., 2011; Rentz et al., 2017). Taken together, these findings support that performance on ART reflects CR.

While there is ample evidence demonstrating that ART provides a good estimate of intelligence and CR at the behavioral level, the neural basis of ART performance is largely unknown. Furthermore, it has not yet been identified whether ART performance reflects the neural function of the regions associated with intelligence or CR. Previous neuroimaging studies on ART focused largely on examining whether CR measured by ART helps to cope against neuropathology in individuals with cognitive impairment (Alexander et al., 1997; Vemuri et al., 2011; Rentz et al., 2017). To our knowledge, however, there are no studies to date that looked directly into the neural substrates of ART performance.

Therefore, the present study aimed to identify the neural correlates of ART performance in cognitively normal (CN) adults. To achieve this aim, the correlation between ART performance and regional cerebral glucose metabolism was examined with brain ^18^F-fluorodeoxyglucose (FDG)-positron emission tomography (PET), which has been known to provide a reliable index of neural activity (Teipel et al., 2006; Melrose et al., 2013; Han et al., 2015). In addition, the neural correlates were reexamined in individuals without a significant level of amyloid-β (Aβ) deposition in the AD signature regions shown on ^11^C-labelled Pittsburgh Compound B (PIB)-PET, in order to eliminate the influence of AD pathology on cerebral glucose metabolism.

## Materials and Methods

### Subjects

This study included 271 healthy CN middle- and old-aged adults who participated in the Korean Brain Aging Study for the Early Diagnosis and Prediction of Alzheimer’s disease (KBASE), which is an ongoing prospective cohort study established in 2014 (Byun et al., 2017). All subjects underwent comprehensive clinical and neuropsychological assessments and multi-modal brain imaging including brain FDG-PET and PiB-PET. Details of the inclusion and exclusion criteria were described previously (Byun et al., 2017). The inclusion criteria for participants with normal cognition were: (a) aged 55–90 years (inclusive); (b) Clinical Dementia Rating score of 0; and (c) no diagnosis of mild cognitive impairment or dementia. Participants who met the following criteria were excluded: (a) any present serious medical, psychiatric, or neurological disorder that could affect mental functioning; (b) presence of severe communication problems that would make a clinical examination or brain scan difficult; (c) contraindications for MRI; (d) absence of a reliable informant; (e) illiteracy; and (f) participation in another clinical trial and treatment with an investigational product.

All subjects provided written informed consent prior to the administration of the study procedure. The study protocol was approved by the Institutional Review Boards of Seoul National University Hospital (C-1401-027-547) and SNU-SMG Boramae Center, Seoul, South Korea (26-2015-60), and was conducted in accordance with the recommendations of the current version of the Declaration of Helsinki.

### Clinical Assessments

All participants received standardized clinical assessments by trained psychiatrists based on the KBASE clinical assessment protocol (Byun et al., 2017) which corresponded with the Korean version of the Consortium to Establish a Registry for AD Assessment Packet (CERAD-K; Lee et al., 2002). In addition, the KBASE neuropsychological assessments (Byun et al., 2017) incorporating the CERAD-K neuropsychological battery (Lee et al., 2004) were administered to all participants by a neuropsychologist or trained psychometrists.

### FDG-PET Acquisition and Preprocessing

FDG-PET scans were performed using a 3.0T Biograph mMR (PET-MR) scanner (Siemens, Germany) and 3D T1-weighted magnetic resonance imaging was simultaneously performed with PET. Details of the acquisition were described previously (Byun et al., 2017).

The FDG-PET data were preprocessed using Statistical Parametric Mapping 12 (SPM12; Institute of Neurology, University College of London, United Kingdom) implemented in Matlab 2015ba (Mathworks, Natick, MA, USA). In the first step, static FDG-PET images were co-registered to individual T1 structural images. Next, transformation parameters were calculated from the individual T1 images that were coregistered to the MNI template image. The forward parameters were used to spatially normalize individual T1 and FDG-PET images to the MNI template. The spatially normalized FDG-PET images were smoothed with a 12-mm Gaussian filter and pons were used as the reference region for intensity normalized (Minoshima et al., 1995).

### PIB-PET Acquisition and Processing

All participants also underwent 3-dimensional (3D) PiB-PET using the same PET-MR machine as the FDG-PET scans. Details of the acquisition were described previously (Byun et al., 2017). Image preprocessing for statistical analyses was performed using SPM 12 implemented in Matlab 2015b. First, the PiB images were coregistered to individual T1 structural images that were coregistered to the MNI template space, and inverse transformation parameters were obtained for spatial normalization. The inverse transformation parameters were used to transform coordinates from the automatic anatomic labeling (AAL) 116 atlas (Tzourio-Mazoyer et al., 2002) into an individual space for each subject (resampling voxel size = 1 × 0.98 × 0.98 mm) with the IBASPM software. The segmented cerebral gray matter segment image from each subject was used to mask the non-gray matter portion of the atlas.

Mean cerebral PiB uptake values were extracted using the individual AAL 116 atlas from the T1-coregistered PiB-PET images and quantitative normalization was performed using the cerebellar gray matter as the reference region (Lopresti et al., 2005). The probabilistic cerebellar atlas (Institute of Cognitive Neuroscience, UCL; Cognitive Neuroscience Laboratory, Royal Holloway) that was transformed into individual space was used to obtain mean cerebellar PiB uptakes from the cerebellar lobular regions except for the vermis. The AAL algorithm (Tzourio-Mazoyer et al., 2002) and a region-combining method (Reiman et al., 2009) were applied to determine regions of interest (ROIs) to characterize the PiB retention level in the frontal, lateral parietal, posterior cingulate-precuneus (PC-PRC), and lateral temporal regions. Each participant was classified as Aβ-positive if the standardized uptake value ratio (SUVR) value was >1.4 in at least one of the four ROIs or as Aβ-negative if the SUVR value of all four ROIs was ≤1.4 (Reiman et al., 2009; Villeneuve et al., 2015).

### The Korean Adult Reading Test (KART)

The KART, the validated Korean version of ART, was administered to all participants (Yi et al., 2017). KART-estimated Wechsler Adult Intelligence Scale, 4th edition (K-WAIS-IV) full-scale IQ (FSIQ) was used as a measure for ART performance.

### Statistical Analyses

Statistical analyses were performed with SPSS 22.0 and SPM12. Correlation between KART-estimated FSIQ score and regional cerebral glucose metabolism was examined using voxel-wise regression with age and gender as covariates. For explorative purposes, the statistical threshold was set at *p* < 0.005 (uncorrected) and a cluster size threshold of 1,062 voxels was applied to correct for multiple comparisons. The cluster size threshold was determined based on a cluster correction procedure in Analysis of Functional and Neural image (i.e., 3dClustSim), with 10,000 iterations of Monte Carlo simulations on anatomical cerebral mask dataset (Forman et al., 1995). The results with the statistical threshold set at *p* < 0.001 (uncorrected) and a cluster size threshold of 527 voxels based on the same cluster correction procedure as the above are presented in the Supplementary Tables S1, S2. In addition, the mean FDG-PET metabolism SUVR values were extracted from the clusters presenting a significant correlation with the KART-estimated FSIQ score. Partial correlation analyses controlling for age and gender were implemented with the FDG-PET SUVR data to examine the strength of the correlations between KART-performance and FDG uptake. The abovementioned analyses were performed again after excluding the Aβ-positive participants.

## Results

### Subject Characteristics

The demographic characteristics of the study sample are summarized in Table 1. The sample consisted of 271 participants, of which 51.7% (*n* = 140) were female and 86.7% (*n* = 235) were Aβ-negative. Participants had a mean age of 69.0 years (SD = 8.1) and average years of education of 11.8 (SD = 4.8). The mean KART error score was 5.0 (SD = 4.9) and the mean FSIQ score estimated from the KART error score was 116.0 (SD = 9.9).

**Table 1:** Demographic characteristics.

Characteristics	CN
*N*	271
Age, mean (SD, range)	00069.0 (8.1, 55–87)
Female, *N* (%)	140 (51.7)
Years of education, mean (SD)	11.8 (4.8)
KART error score, mean (SD)	5.0 (4.9)
KART-estimated FSIQ, mean (SD)	116.0 (9.9)
Aβ-negative, *N* (%)	235 (86.7)

*Values are mean (SD) or count. SD, standard deviation; N, count; KART-estimated FSIQ, Wechsler Adult Intelligence Scale Full-Scale IQ estimated using the KART; Aβ, amyloid-beta*.

### Correlation Between Regional Cerebral Glucose Metabolism and KART Performance in all CN Subjects

**Figure 1:**
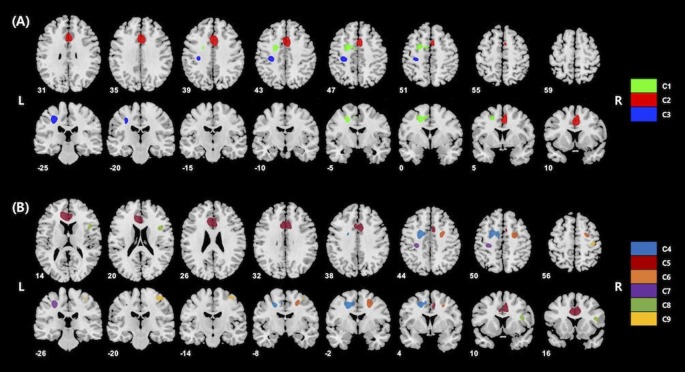
**(A)** Results of whole-brain voxel-wise analysis presenting significant associations between cerebral glucose metabolism and korean adult reading test (KART)-estimated full-scale IQ (FSIQ) score in all cognitively normal (CN) subjects. Positive correlations were shown in the left middle frontal gyrus (MFG), right anterior cingulate gyrus (ACG) and left postcentral gyrus (PCG). Cluster numbers (e.g., C1) correspond to those in Table 2. **(B)** Results of whole-brain voxel-wise analysis presenting significant associations between cerebral glucose metabolism and KART-estimated FSIQ score in amyloid-β (Aβ)-negative CN subjects. Positive correlations were shown in the bilateral middle frontal gyri, bilateral anterior cingulate gyri, bilateral postcentral gyri and right inferior frontal gyrus (IFG). Cluster numbers correspond to those in Table 3.

The voxel-wise analysis using age and gender as nuisance covariates revealed significant positive correlations between KART-estimated FSIQ score and regional cerebral glucose metabolism in the frontal and primary somatosensory regions, particularly in the left middle frontal gyrus (MFG), right anterior cingulate gyrus (ACG) and left postcentral gyrus (PCG; Table 2, Figure 1A).

**Table 2:** Positive correlations between regional cerebral glucose metabolism and The Korean Adult Reading Test (KART)-estimated full-scale IQ (FSIQ) score after adjusting for age and gender in all cognitively normal (CN) subjects.

Regions	BA	Coordinates (mm)			Extent voxels	*T*-value	Cluster number
		*x*	*y*	*z*			
L middle frontal gyrus	6/9	−21	−1	46	1,990	3.57	C1
R anterior cingulate gyrus	32	5	8	43	5,623	3.38	C2
L postcentral gyrus	3	−30	−25	46	1,545	3.35	C3

*p < 0.005 (uncorrected) with significance of *k* > 1,062. Adjusted for age and gender. Coordinates are in Montreal Neurological Institute (MNI) space. Cluster numbers are labeled for each cluster (i.e., C1 denotes cluster no.1). BA, approximate Brodmann area; L, left hemisphere; R, right hemisphere*.

**Figure 2:**
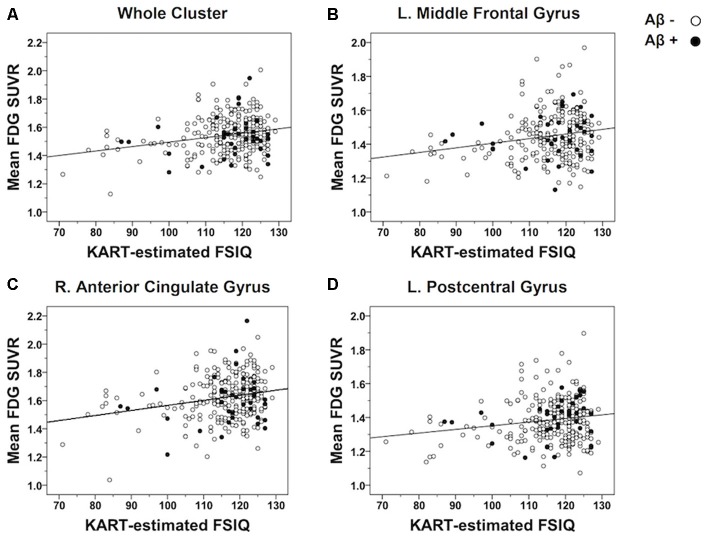
Scatterplots demonstrating the strength of correlations between KART-estimated FSIQ score and regional glucose metabolism in all CN subjects. Mean FDG-PET SUVR values were extracted from the clusters of voxels showing a significant association with KART performance and examined the correlation with KART performance with using a partial correlation analysis adjusted for age and gender. There were positive significant correlations between KART-estimated IQ score and mean FDG-PET SUVR values in all clusters: **(A)** whole cluster; **(B)** left middle frontal gyrus; **(C)** right anterior cingulate gyrus; **(D)** left postcentral gyrus.

In order to further examine the strength of the correlations, raw mean FDG-PET SUVR values were extracted from each cluster showing a significant association with the KART performance and partial correlation analyses were performed controlling for the effects of age and gender. There were positive and moderately significant correlations between KART-estimated IQ score and mean FDG-PET SUVR values in all clusters (whole cluster: *r* = 0.21, *p* = 0.001; left MFG: *r* = 0.20, *p* = 0.001; right ACG: *r* = 0.19, *p* = 0.002; left PCG: *r* = 0.18, *p* = 0.003; Figure 2).

### Correlation Between Regional Cerebral Glucose Metabolism and KART Performance in Aβ-Negative CN Subjects

For Aβ-negative subsample (*n* = 235), the voxel-wise analysis showed positive correlations in more widespread clusters of the bilateral frontal and primary somatosensory regions including the bilateral MFC, bilateral ACG, bilateral PCG and right inferior frontal gyrus (IFG; Table 3, Figure 1B). Particularly, additional positive correlations were found in the right lateral frontal cortex (LFC; middle and inferior frontal gyri), left ACG and right PCG after Aβ-positive subjects were excluded.

**Table 3:** Positive correlations between regional cerebral glucose metabolism and KART-estimated FSIQ score after adjusting for age and gender in Aβ-negative CN subjects.

Regions	BA	Coordinates (mm)			Extent voxels	*T*-value	Cluster number
		*x*	*y*	*z*			
L middle frontal gyrus	6,8	−21	−2	45	3,071	3.66	C4
L anterior cingulate gyrus	32	−6	37	9	14,444	3.52	C5
R middle frontal gyrus	6	23	−4	48	1,878	3.38	C6
L postcentral gyrus	3	−29	−25	47	1,194	3.21	C7
R anterior cingulate gyrus	32	6	16	34	14,444	3.19	C5
R inferior frontal gyrus	9	42	12	19	1,092	3.01	C8
R postcentral gyrus	3	38	−20	60	1,543	2.95	C9

*p < 0.005 (uncorrected) with significance of k > 1,062. Adjusted for age, and gender. Coordinates are in Montreal Neurological Institute (MNI) space. Cluster numbers are labeled for each cluster (i.e., C1 denotes cluster no.1). BA, approximate Brodmann area; L, left hemisphere; R, right hemisphere*.

Partial correlation analyses adjusted for the effects of age and gender were repeated to see the strengths of the correlations with the raw mean FDG-PET SUVR values from these significant clusters and the KART performance. Relatively modest and significant correlations were found in all clusters (whole cluster: *r* = 0.23, *p* = 0.001; right IFG: *r* = 0.19, *p* = 0.004; left MFG: *r* = 0.21, *p* = 0.001; right MFG: *r* = 0.20, *p* = 0.002; left ACG: *r* = 0.21, *p* = 0.001; right ACG: *r* = 0.21, *p* = 0.001; left PCG: *r* = 0.19, *p* = 0.003; right PCG: *r* = 0.19, *p* = 0.004; Supplementary Figure S1).

## Discussion

The current study identified the brain regions correlated with ART performance by examining the associations between resting-state glucose metabolism and KART scores using a voxel-wise approach in CN adults. To sum up, in the whole sample including individuals both with and without significant levels of Aβ deposition, KART performance was positively correlated with regional glucose metabolism in the frontal and primary somatosensory areas—more specifically the left MFG, right ACG, and left PCG. After excluding Aβ-positive subjects, positive correlations with KART performance were found in much larger areas of the frontal and primary somatosensory regions including the bilateral MFG, right IFG, bilateral ACG and bilateral PCG. These findings suggest that the neural substrates of ART performance are located primarily in the frontal and primary somatosensory areas.

These results emphasize the involvement of the frontal and primary somatosensory areas in premorbid function, as estimated by ART. The patterns of brain metabolic impairment typical of AD begins in the precuneus and posterior cingulate cortex, spreads to parieto-temporal regions, and finally progresses to frontal and sensory cortices (Mosconi et al., 2007, 2008). The frontal and primary somatosensory regions have been identified as being typically affected in the late stages of AD (Braak and Braak, 1991; Mosconi et al., 2007, 2008). Indeed, the regional neural functions that are found to be associated with KART performance are relatively less vulnerable in AD. Therefore, it may be posited that ART can predict premorbid function by reflecting the neuronal functions of regions that are relatively preserved in AD.

The largest region that showed positive correlations between glucose metabolism and KART performance was the frontal area, particularly the ACC and LFC. The ACC and LFC are components of the frontoparietal network (FPN), which has been reported to be the neural substrates of general intelligence (Margulies et al., 2007; Cole et al., 2012, 2015). The ACC, in particular, is known to support intelligence by handling and processing conflicting streams of information (Botvinick et al., 1999; Cohen et al., 2005; Brown, 2009). In addition to being recognized as a core region for intelligence, the LFC is functionally responsible for regulating the flow and integration of information between other regions (Cole et al., 2012, 2013, 2015).

The postcentral gyri, also known as the primary somatosensory cortex (SSC), were significantly associated with KART performance. While the roles of the SSC pertaining to intelligence are less clear, previous studies showed positive associations between general intelligence and cortical thickness or gray matter volumes of the SSC (Colom et al., 2009, 2013; Karama et al., 2009). Given that the SSC is involved in not only the processing of somatosensory input but also in the integration of sensory and motor signals (Borich et al., 2015), it is therefore possible that this region supports intelligence by working in tandem with the frontal area towards high-level cognitive functions such as perceptual decision making (Colom et al., 2009, 2013; Santarnecchi et al., 2015). While the exact underlying mechanism for the involvement of SSC when performing ART is unclear, the current findings demonstrate that the same SSC region associated with general intelligence is also associated with the proxy for general intelligence.

Notably, the LFC has also been suggested as a putative neural substrate of CR, in addition to being associated with general intelligence. The neural mechanism underlying CR is largely unclear, but several possible mechanisms have been postulated including the task-invariant networks (TIN; Stern et al., 2008; Stern, 2009, 2017). The TIN, which remains active throughout multiple tasks with various levels of processing demands, has been suggested to support CR by serving as a compensatory neural network against brain pathology (Stern, 2006, 2009, 2017). The LFC has been reported to have strong connectivity with multiple TINs and is considered as the hub region of the TINs (Cole et al., 2015; Franzmeier et al., 2017b,c). Given that the TIN is reported to support CR and the hub of the TIN is thought to reside in the LFC, it can be posited that the LFC subserves CR (Franzmeier et al., 2017a,b,c). A recent resting-state fMRI study, which showed that the association between AD-related biomarkers and lower memory performance was attenuated by the global functional connectivity of the LFC in patients with mild cognitive impairment, provides support for the current findings (Franzmeier et al., 2017b). Taken together, the current findings further corroborate the possible role of the LFC—the region most significantly associated with KART performance—as the neural substrate of CR.

An additional interesting finding of the current study is that the regions associated with KART performance became larger when individuals with high levels of Aβ were excluded in the analysis. Considering the fact that accumulated AD pathology generates structural and functional changes in the brain (Perl, 2010; Beason-Held et al., 2013; Mattsson et al., 2014), the neural correlates related to ART performance may vary due to AD pathology, as also observed in the current analyses. Such variation (i.e., the inclusion of much broader regions of functional activity after excluding Aβ-positive subjects) may signify several points. First, this demonstrates that the neuronal functions may be affected by AD neuropathology even in individuals without clinical manifestation of AD. Second, it may indicate that the broader regions found after eliminating Aβ-positive subjects are a more precise representation of the neural correlates of ART performance. Last, given that the associations of the left LFC, left PCG and right ACC—the regions found in the initial analyses with the entire sample—persisted even when those with AD pathology were included, it can be inferred that ART performance is indeed resistant against AD.

In conclusion, this is the first functional neuroimaging study describing the neural substrates associated with ART performance. The positive correlation between ART performance and glucose metabolism in the frontal and primary somatosensory areas indeed corresponds to regions that have been previously reported to be associated with general intelligence and cognitive reserve. The current study provides support, at the neural level, for the use of ART as a measure of premorbid functioning in both research and clinical settings. Moreover, the identification of the neural correlates of ART performance helps to gain an understanding of the neural mechanism underlying CR. Future studies using a multimodal approach such as combining FDG-PET with task-fMRI will provide more detailed information regarding the functional neural substrates that underlie ART performance. Furthermore, future studies utilizing more advanced atlases than AAL will be informative.

## Data Availability Statement

The datasets generated for this study are available on request to the corresponding author.

## Ethics Statement

The studies involving human participants were reviewed and approved by Institutional Review Boards of Seoul National University Hospital (IRB no. C-1401-027-547) and SNU-SMG Boramae Center, Seoul, South Korea (IRB no. 26-2015-60). The patients/participants provided their written informed consent to participate in this study.

## Author Contributions

YL, DY, and DL designed the study, acquired and interpreted the data, and were major contributors to the writing of the manuscript and critically revising the manuscript for intellectual content. ES, JH, HJ, MB, JL, and JJ acquired and analyzed the data and helped to draft the manuscript. DL served as the principal investigator and supervised the study. All authors read and approved the final manuscript.

## Conflict of Interest

The authors declare that the research was conducted in the absence of any commercial or financial relationships that could be construed as a potential conflict of interest.

## References

[B1] Alexander G. E. Furey M. L. Grady C. L. Pietrini P. Brady D. R. Mentis M. J.. (1997). Association of premorbid intellectual function with cerebral metabolism in Alzheimer’s disease: implications for the cognitive reserve hypothesis. Am. J. Psychiatry 154, 165–172. 10.1176/ajp.154.2.165 9016263

[B2] Beason-Held L. L. Goh J. O. An Y. Kraut M. A. O’Brien R. J. Ferrucci L.. (2013). Changes in brain function occur years before the onset of cognitive impairment. J. Neurosci. 33, 18008–18014. 10.1523/JNEUROSCI.1402-13.2013 24227712 PMC3828456

[B3] Borich M. R. Brodie S. M. Gray W. A. Ionta S. Boyd L. A. (2015). Understanding the role of the primary somatosensory cortex: opportunities for rehabilitation. Neuropsychologia 79, 246–255. 10.1016/j.neuropsychologia.2015.07.007 26164474 PMC4904790

[B4] Botvinick M. Nystrom L. E. Fissell K. Carter C. S. Cohen J. D. (1999). Conflict monitoring versus selection-for-action in anterior cingulate cortex. Nature 402, 179–181. 10.1038/46035 10647008

[B5] Braak H. Braak E. (1991). Neuropathological stageing of Alzheimer-related changes. Acta Neuropathol. 82, 239–259. 10.1007/bf00308809 1759558

[B6] Brown J. W. (2009). Conflict effects without conflict in anterior cingulate cortex: multiple response effects and context specific representations. NeuroImage 47, 334–341. 10.1016/j.neuroimage.2009.04.034 19375509 PMC2709509

[B7] Byun M. S. Yi D. Lee J. H. Choe Y. M. Sohn B. K. Lee J. Y.. (2017). Korean brain aging study for the early diagnosis and prediction of Alzheimer’s disease: methodology and baseline sample characteristics. Psychiatry Investig. 14, 851–863. 10.4306/pi.2017.14.6.851 29209391 PMC5714729

[B8] Cohen M. X. Heller A. S. Ranganath C. (2005). Functional connectivity with anterior cingulate and orbitofrontal cortices during decision-making. Cogn. Brain Res. 23, 61–70. 10.1016/j.cogbrainres.2005.01.010 15795134

[B9] Cole M. W. Ito T. Braver T. S. (2015). Lateral prefrontal cortex contributes to fluid intelligence through multinetwork connectivity. Brain Connect. 5, 497–504. 10.1089/brain.2015.0357 26165732 PMC4601676

[B10] Cole M. W. Reynolds J. R. Power J. D. Repovs G. Anticevic A. Braver T. S. (2013). Multi-task connectivity reveals flexible hubs for adaptive task control. Nat. Neurosci. 16, 1348–1355. 10.1038/nn.3470 23892552 PMC3758404

[B11] Cole M. W. Yarkoni T. Repovs G. Anticevic A. Braver T. S. (2012). Global connectivity of prefrontal cortex predicts cognitive control and intelligence. J. Neurosci. 32, 8988–8999. 10.1523/JNEUROSCI.0536-12.2012 22745498 PMC3392686

[B12] Colom R. Burgaleta M. Roman F. J. Karama S. Alvarez-Linera J. Abad F. J.. (2013). Neuroanatomic overlap between intelligence and cognitive factors: morphometry methods provide support for the key role of the frontal lobes. NeuroImage 72, 143–152. 10.1016/j.neuroimage.2013.01.032 23357078

[B13] Colom R. Haier R. J. Head K. Álvarez-Linera J. Quiroga M. Á. Shih P. C. (2009). Gray matter correlates of fluid, crystallized, and spatial intelligence: testing the P-FIT model. Intelligence 37, 124–135. 10.1016/j.intell.2008.07.007

[B14] Crawford J. R. Millar J. Milne A. B. (2001). Estimating premorbid IQ from demographic variables: a comparison of a regression equation vs. clinical judgement. Br. J. Clin. Psychol. 40, 97–105. 10.1348/014466501163517 11317952

[B15] Forman S. D. Cohen J. D. Fitzgerald M. Eddy W. F. Mintun M. A. Noll D. C. (1995). Improved assessment of significant activation in functional magnetic resonance imaging (fMRI): use of a cluster-size threshold. Magn. Reson. Med. 33, 636–647. 10.1002/mrm.1910330508 7596267

[B16] Franzmeier N. Buerger K. Teipel S. Stern Y. Dichgans M. Ewers M.. (2017a). Cognitive reserve moderates the association between functional network anti-correlations and memory in MCI. Neurobiol. Aging 50, 152–162. 10.1016/j.neurobiolaging.2016.11.013 28017480

[B17] Franzmeier N. Duering M. Weiner M. Dichgans M. Ewers M. Alzheimer’s Disease Neuroimaging Initiative (ADNI). (2017b). Left frontal cortex connectivity underlies cognitive reserve in prodromal Alzheimer disease. Neurology 88, 1054–1061. 10.1212/wnl.0000000000003711 28188306 PMC5384837

[B18] Franzmeier N. Göttler J. Grimmer T. Drzezga A. Áraque-Caballero M. A. Simon-Vermot L.. (2017c). Resting-state connectivity of the left frontal cortex to the default mode and dorsal attention network supports reserve in mild cognitive impairment. Front. Aging Neurosci. 9:264. 10.3389/fnagi.2017.00264 28824423 PMC5545597

[B19] Han J. Y. Byun M. S. Seo E. H. Yi D. Choe Y. M. Sohn B. K.. (2015). Functional neural correlates of figure copy and recall task performances in cognitively impaired individuals: an 18F-FDG-PET study. Neuroreport 26, 1077–1082. 10.1097/wnr.0000000000000476 26509549

[B20] Karama S. Ad-Dab’bagh Y. Haier R. Deary I. Lyttelton O. Lepage C.. (2009). Positive association between cognitive ability and cortical thickness in a representative US sample of healthy 6 to 18 year-olds. Intelligence 37, 145–155. 10.1016/j.intell.2008.09.006 20161325 PMC2678742

[B22] Lee J. H. Lee K. U. Lee D. Y. Kim K. W. Jhoo J. H. Kim J. H.. (2002). Development of the korean version of the consortium to establish a registry for Alzheimer’s disease assessment packet (CERAD-K): clinical and neuropsychological assessment batteries. J. Gerontol. B Psychol. Sci. Soc. Sci. 57, P47–P53. 10.1093/geronb/57.1.p47 11773223

[B21] Lee D. Y. Lee K. U. Lee J. H. Kim K. W. Jhoo J. H. Kim S. Y.. (2004). A normative study of the CERAD neuropsychological assessment battery in the Korean elderly. J. Int. Neuropsychol. Soc. 10, 72–81. 10.1017/s1355617704101094 14751009

[B23] Lezak M. D. Howieson D. B. Loring D. W. Hannay H. J. Fischer J. S. (2004). Neuropsychological Assessment. Oxford; New York, NY: Oxford University Press.

[B24] Lo R. Y. Jagust W. J. Alzheimer’s Disease Neuroimaging Initiative (2013). Effect of cognitive reserve markers on Alzheimer pathologic progression. Alzheimer Dis. Assoc. Disord. 27, 343–350. 10.1097/WAD.0b013e3182900b2b 23552443 PMC3745532

[B25] Lopresti B. J. Klunk W. E. Mathis C. A. Hoge J. A. Ziolko S. K. Lu X.. (2005). Simplified quantification of Pittsburgh Compound B amyloid imaging PET studies: a comparative analysis. J. Nucl. Med. 46, 1959–1972. 10.1038/sj.jcbfm.9591524.0589 16330558

[B26] Margulies D. S. Kelly A. M. Uddin L. Q. Biswal B. B. Castellanos F. X. Milham M. P. (2007). Mapping the functional connectivity of anterior cingulate cortex. NeuroImage 37, 579–588. 10.1016/j.neuroimage.2007.05.019 17604651

[B27] Matsuoka K. Uno M. Kasai K. Koyama K. Kim Y. (2006). Estimation of premorbid IQ in individuals with Alzheimer’s disease using Japanese ideographic script (Kanji) compound words: japanese version of national adult reading test. Psychiatry Clin. Neurosci. 60, 332–339. 10.1111/j.1440-1819.2006.01510.x 16732750

[B28] Mattsson N. Insel P. S. Nosheny R. Tosun D. Trojanowski J. Q. Shaw L. M.. (2014). Emerging β-amyloid pathology and accelerated cortical atrophy. JAMA Neurol. 71, 725–734. 10.1001/jamaneurol.2014.446 24781145 PMC4410966

[B29] McGurn B. Starr J. Topfer J. Pattie A. Whiteman M. Lemmon H.. (2004). Pronunciation of irregular words is preserved in dementia, validating premorbid IQ estimation. Neurology 62, 1184–1186. 10.1212/01.wnl.0000103169.80910.8b 15079021

[B30] Melrose R. J. Harwood D. Khoo T. Mandelkern M. Sultzer D. L. (2013). Association between cerebral metabolism and Rey-Osterrieth Complex Figure Test performance in Alzheimer’s disease. J. Clin. Exp. Neuropsychol. 35, 246–258. 10.1080/13803395.2012.763113 23387510 PMC3727972

[B31] Minoshima S. Frey K. A. Foster N. L. Kuhl D. E. (1995). Preserved pontine glucose metabolism in Alzheimer disease: a reference region for functional brain image (PET) analysis. J. Comput. Assist. Tomogr. 19, 541–547. 10.1097/00004728-199507000-00006 7622680

[B32] Mosconi L. Brys M. Glodzik-Sobanska L. De Santi S. Rusinek H. de Leon M. J. (2007). Early detection of Alzheimer’s disease using neuroimaging. Exp. Gerontol. 42, 129–138. 10.1016/j.exger.2006.05.016 16839732

[B33] Mosconi L. Tsui W. H. Herholz K. Pupi A. Drzezga A. Lucignani G.. (2008). Multicenter standardized 18F-FDG PET diagnosis of mild cognitive impairment, Alzheimer’s disease, and other dementias. J. Nucl. Med. 49, 390–398. 10.2967/jnumed.107.045385 18287270 PMC3703818

[B34] Nelson H. E. Willison J. (1991). National Adult Reading Test (NART). Windsor, UK: Nfer-Nelson.

[B35] Perl D. P. (2010). Neuropathology of Alzheimer’s disease. Mt Sinai J. Med. 77, 32–42. 10.1002/msj.20157 20101720 PMC2918894

[B36] Reiman E. M. Chen K. Liu X. Bandy D. Yu M. Lee W.. (2009). Fibrillar amyloid-β burden in cognitively normal people at 3 levels of genetic risk for Alzheimer’s disease. Proc. Natl. Acad. Sci. U S A 106, 6820–6825. 10.1073/pnas.0900345106 19346482 PMC2665196

[B37] Rentz D. M. Mormino E. C. Papp K. V. Betensky R. A. Sperling R. A. Johnson K. A. (2017). Cognitive resilience in clinical and preclinical Alzheimer’s disease: the association of amyloid and tau burden on cognitive performance. Brain Imaging Behav. 11, 383–390. 10.1007/s11682-016-9640-4 27738998 PMC5391311

[B38] Santarnecchi E. Tatti E. Rossi S. Serino V. Rossi A. (2015). Intelligence-related differences in the asymmetry of spontaneous cerebral activity. Hum. Brain Mapp. 36, 3586–3602. 10.1002/hbm.22864 26059228 PMC6868935

[B39] Sasanuma S. Sakuma N. Kitano K. (1992). Reading kanji without semantics: evidence from a longitudinal study of dementia. Cogn. Neuropsychol. 9, 465–486. 10.1080/02643299208252068

[B40] Snowdon D. A. Kemper S. J. Mortimer J. A. Greiner L. H. Wekstein D. R. Markesbery W. R. (1996). Linguistic ability in early life and cognitive function and Alzheimer’s disease in late life. Findings from the Nun Study. JAMA 275, 528–532. 10.1001/jama.1996.03530310034029 8606473

[B41] Stern Y. (2006). Cognitive reserve and Alzheimer disease. Alzheimer Dis. Assoc. Disord. 20, 112–117. 10.1097/01.wad.0000213815.20177.19 16772747

[B42] Stern Y. (2009). Cognitive reserve. Neuropsychologia 47, 2015–2028. 10.1016/j.neuropsychologia.2009.03.004 19467352 PMC2739591

[B43] Stern Y. (2017). An approach to studying the neural correlates of reserve. Brain Imaging Behav. 11, 410–416. 10.1007/s11682-016-9566-x 27450378 PMC5810375

[B44] Stern Y. Zarahn E. Habeck C. Holtzer R. Rakitin B. C. Kumar A.. (2008). A common neural network for cognitive reserve in verbal and object working memory in young but not old. Cereb. Cortex 18, 959–967. 10.1093/cercor/bhm134 17675368 PMC2519015

[B45] Stern Y. Zarahn E. Hilton H. J. Flynn J. DeLaPaz R. Rakitin B. (2003). Exploring the neural basis of cognitive reserve. J. Clin. Exp. Neuropsychol. 25, 691–701. 10.1076/jcen.25.5.691.14573 12815506

[B46] Teipel S. J. Willoch F. Ishii K. Bürger K. Drzezga A. Engel R.. (2006). Resting state glucose utilization and the CERAD cognitive battery in patients with Alzheimer’s disease. Neurobiol. Aging 27, 681–690. 10.1016/j.neurobiolaging.2005.03.015 15908048

[B47] Tzourio-Mazoyer N. Landeau B. Papathanassiou D. Crivello F. Etard O. Delcroix N.. (2002). Automated anatomical labeling of activations in SPM using a macroscopic anatomical parcellation of the MNI MRI single-subject brain. NeuroImage 15, 273–289. 10.1006/nimg.2001.0978 11771995

[B48] Vemuri P. Weigand S. D. Przybelski S. A. Knopman D. S. Smith G. E. Trojanowski J. Q.. (2011). Cognitive reserve and Alzheimer’s disease biomarkers are independent determinants of cognition. Brain 134, 1479–1492. 10.1093/brain/awr049 21478184 PMC3097887

[B49] Villeneuve S. Rabinovici G. D. Cohn-Sheehy B. I. Madison C. Ayakta N. Ghosh P. M.. (2015). Existing Pittsburgh Compound-B positron emission tomography thresholds are too high: statistical and pathological evaluation. Brain 138, 2020–2033. 10.1093/brain/awv112 25953778 PMC4806716

[B50] Whalley L. J. Deary I. J. (2001). Longitudinal cohort study of childhood IQ and survival up to age 76. BMJ 322:819. 10.1136/bmj.322.7290.819 11290633 PMC30556

[B51] Yeo R. A. Arden R. Jung R. E. (2011). Alzheimer’s disease and intelligence. Curr. Alzheimer Res. 8, 345–353. 10.2174/156720511795745276 21222590

[B52] Yi D. Seo E. H. Han J. Y. Sohn B. K. Byun M. S. Lee J. H.. (2017). Development of the Korean Adult Reading Test (KART) to estimate premorbid intelligence in dementia patients. PLoS One 12:e0181523. 10.1371/journal.pone.0181523 28723964 PMC5517066

