# Supplementary material for: Resting State Glucose Utilization and Adult Reading Test Performance
Source: Front Aging Neurosci. 2020 Mar 5;12:48. doi: 10.3389/fnagi.2020.00048 (PMC7066080; doi:10.3389/fnagi.2020.00048)
Supplement: Supplementary file 1 [file Presentation_1.pptx]

## Slide 1
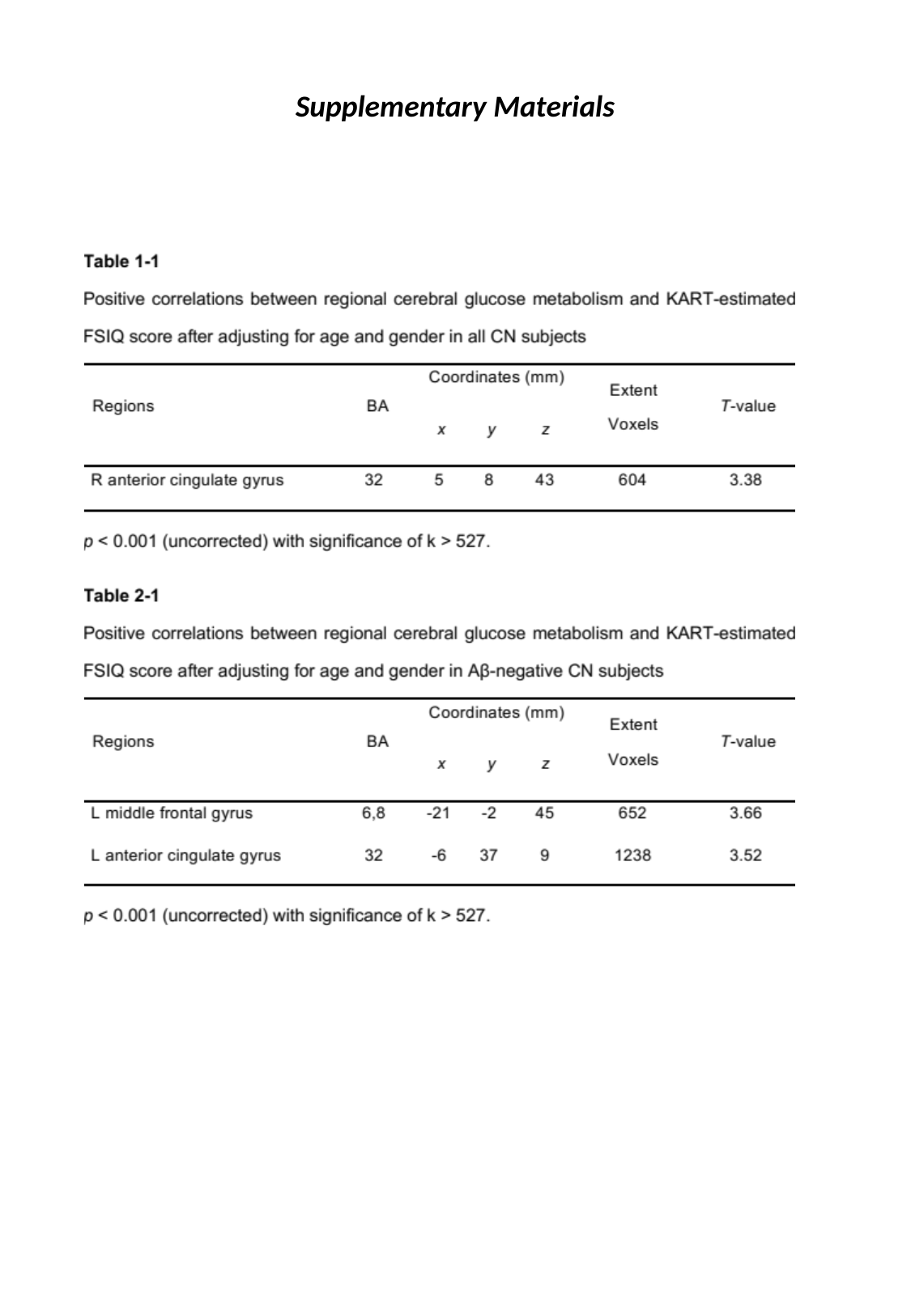

Supplementary Materials

## Slide 2
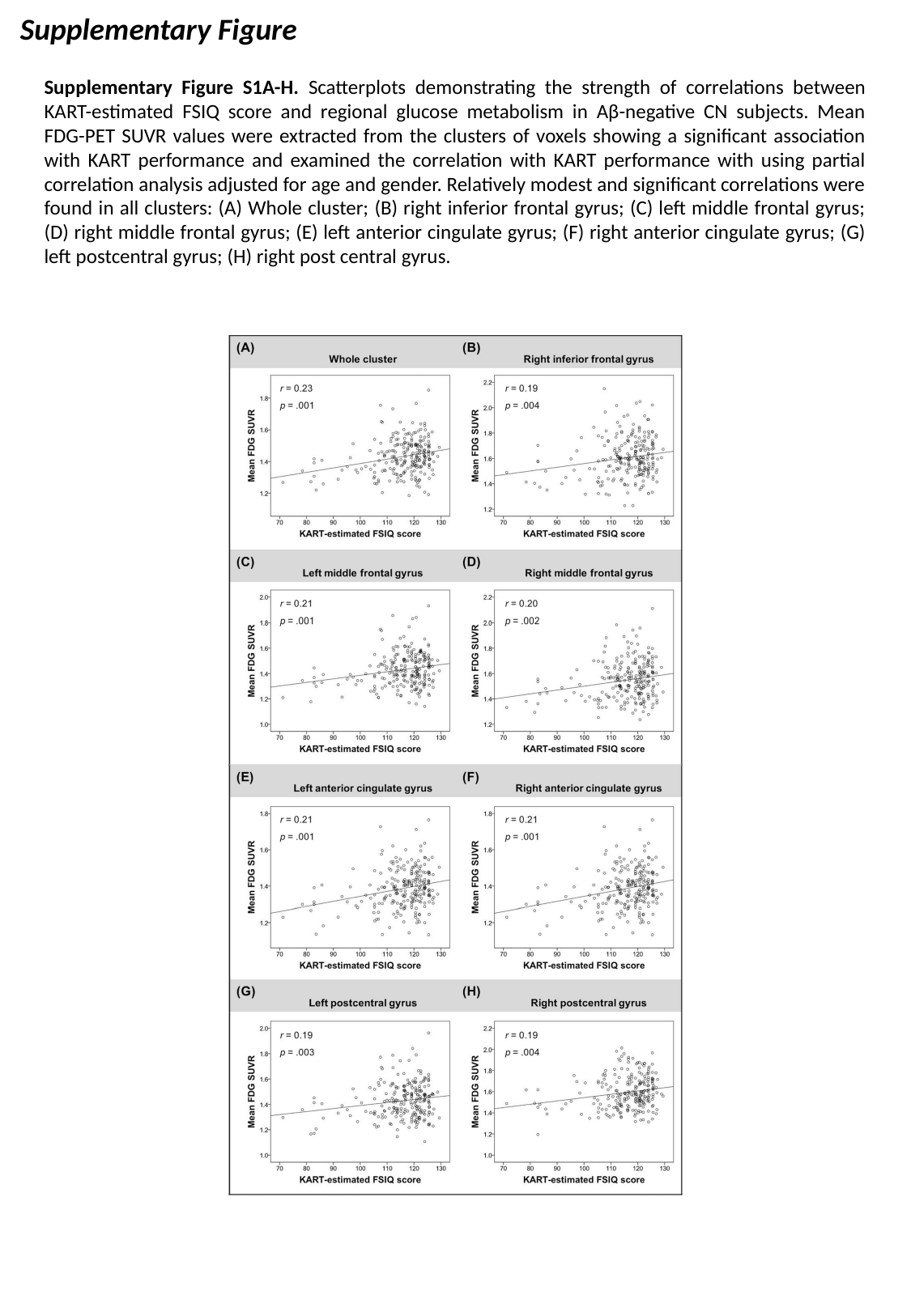

Supplementary Figure
Supplementary Figure S1A-H. Scatterplots demonstrating the strength of correlations between KART-estimated FSIQ score and regional glucose metabolism in Aβ-negative CN subjects. Mean FDG-PET SUVR values were extracted from the clusters of voxels showing a significant association with KART performance and examined the correlation with KART performance with using partial correlation analysis adjusted for age and gender. Relatively modest and significant correlations were found in all clusters: (A) Whole cluster; (B) right inferior frontal gyrus; (C) left middle frontal gyrus; (D) right middle frontal gyrus; (E) left anterior cingulate gyrus; (F) right anterior cingulate gyrus; (G) left postcentral gyrus; (H) right post central gyrus.
